# Lateral Periodontal Cyst Treated with Enucleation and Guided Bone Regeneration: A Report of a Case and a Review of Pertinent Literature

**DOI:** 10.1155/2019/4591019

**Published:** 2019-07-08

**Authors:** Sundar Ramalingam, Yasser Fahad Alrayyes, Khalid Buayjan Almutairi, Ibrahim O. Bello

**Affiliations:** ^1^Department of Oral and Maxillofacial Surgery, College of Dentistry, King Saud University, Riyadh 11545, Saudi Arabia; ^2^Dental University Hospital, College of Dentistry, King Saud University, Riyadh 11545, Saudi Arabia; ^3^Department of Oral Medicine and Diagnostic Sciences, College of Dentistry, King Saud University, Riyadh 11545, Saudi Arabia

## Abstract

Lateral periodontal cyst (LPC) is an uncommon developmental odontogenic cyst arising on the lateral surface of tooth roots. Commonly reported in mandibular canine-premolar or maxillary anterior regions, it presents as a well-circumscribed or tear drop-shaped radiolucency with a sclerotic border. Associated teeth are asymptomatic and vital, and roots may be displaced without resorption. Histopathologically, cystic lining resembles reduced enamel epithelium along with glycogen-rich clear cells and epithelial plaques. Unilateral variant of LPC has low recurrence and is managed by enucleation. A 43-year-old male patient reported with asymptomatic swelling in the left mandibular canine and first premolar region. Both teeth were vital, and radiographs revealed well-circumscribed radiolucency between the roots. Following consent, surgical enucleation and guided bone regeneration (GBR) with xenograft and resorbable collagen membrane were done under local anesthesia. The immediate postoperative period was uneventful, and complete bone fill of cystic cavity and healing of periodontal tissues was observed after a one-year follow-up. Histopathologic examination confirmed the diagnosis. LPC should be a differential diagnosis in cystic lesions lateral to the surface of a tooth and without any associated inflammation. Based on this case report, unicystic LPC can be successfully managed through surgical enucleation with GBR for better periodontal healing.

## 1. Introduction

Lateral periodontal cyst (LPC) is a relatively rare odontogenic pathology, representing about 0.4% of all odontogenic cysts [1–4]. It has been reported predominantly as an incidental finding and is a harmless entity characterized by an intraosseous, nonkeratinized, noninflammatory, and developmental odontogenic cyst of epithelial origin [2, 4, 5]. LPC is usually symptomless and is discovered during routine radiographic examination and occurs most frequently in the alveolar process of the mandibular canine and premolar regions, followed by the anterior maxilla [5]. Although it is commonly reported between the fourth to the seventh decades of life, cases have been reported in younger individuals too and it does not have a specific gender predilection [2]. Interestingly the epithelial cell of origin of LPC has been debated considerably [2]. Reports from the literature indicate a possible origin from either the rests of the dental lamina, reduced enamel epithelium, or rests of Malassez [3, 5, 6].

According to the World Health Organization (WHO) classification of odontogenic tumors and cysts (4th Edition, 2017), LPC is classified as an odontogenic cyst of developmental origin (Table 1) [7]. Clinically, LPC presents as an asymptomatic gingival swelling occurring on the facial aspect in between two teeth, and it should be differentiated from its soft tissue counterpart, the gingival cyst of adults (GCA), which presents as a blue fluctuant swelling [1, 5, 8]. Radiologically, they present as a well-circumscribed unilocular radiolucency with a sclerotic margin, in the alveolar process, in between the roots of vital teeth [5, 8]. While most LPCs are less than 1 cm in diameter, the uncommon botryoid variant may present as a larger and multilocular radiolucency, extending up to the periapical region [1, 5, 9]. Described for the first time in 1973, the botryoid odontogenic cyst (BOC) is associated with a greater risk of recurrence (30.1%), in comparison to LPC [9]. Based on reports available in the literature, BOC is known to affect patients in the fifth decade, with a slight preponderance towards females (53.5%) and more commonly in the mandible (83.3%) [4, 9]. Interestingly, LPC, BOC, and GCA have a common histogenetic origin and are classified as developmental odontogenic cysts as per WHO classification [4, 7]. It is reported that BOC arises from multiple foci of closely located epithelial rests, resulting in a multicystic lesion [4, 9]. Nevertheless, multiple, separate LPCs occurring in close proximity have also been reported by Siponen et al. [4].

Although untreated odontogenic cysts could cause root resorption in adjacent teeth, it has not been reported in association with LPC [1, 8]. Moreover, the associated teeth are usually vital unless they have been otherwise affected by dental caries or periodontitis [2]. Pain and tenderness, with or without cortical expansion in cases of LPC, have also been infrequently reported in the literature [1, 8]. LPCs are commonly differentially diagnosed as either a gingival cyst, an odontogenic keratocyst, or a residual cyst owing to their clinical and radiographic similarities [2, 5]. However, LPCs are histologically unique and are most frequently lined by a thin, nonkeratinizing layer of squamous or cuboidal epithelium, typically ranging from 1 to 3 cell layers in thickness and resembling the reduced enamel epithelium [2, 4, 5, 10, 11]. The epithelial cells in an LPC present with small, pyknotic nuclei and are occasionally separated by intercellular fluid [1, 4, 8]. In addition, focal plaques of epithelial thickenings in a fusiform or spindle-shaped pattern, along with glycogen-rich clear cells, have also been reported in the cystic lining [4, 10].

Treatment of LPC, which provided that the lesion is unilocular on radiological examination, is by conservative surgical enucleation and following up the patient over a period of six months to one year, in order to monitor for recurrence [1, 12]. Nevertheless, simple surgical enucleation is associated with a residual bone defect which could potentially affect the periodontal health of the associated teeth [10]. With the advent of tissue regeneration techniques in dentistry, several techniques including guided tissue regeneration (GTR) and guide bone regeneration (GBR) have been reported for the treatment of bone defects, following enucleation of LPC [10, 13]. The technique of GBR, which has been reported with a considerable clinical success, involves placement of an osteoconductive scaffold within a bone defect and covering it with a resorbable or nonresorbable barrier membrane [14].

The aim of this paper is to present a case of LPC treated with surgical enucleation and primary bone defect repair using GBR with xenograft and resorbable collagen membrane, along with a recurrence-free follow-up period of one year. In addition, this case is being reported for the similarities in clinical and radiographic presentation between LPC and other odontogenic lesions and a proposed modality towards definitive treatment.

## 2. Case Presentation

A 43-year-old Saudi male patient reported to the oral surgery outpatient clinic at the College of Dentistry and Dental University Hospital, King Saud University, with a complaint of painless swelling in the left mandibular premolar region. Although the history revealed a presence of the swelling for more than one year without any associated symptoms, the patient preferred to obtain clinical consultation as he was concerned about the swelling. The patient had no previous history of dental treatment except for periodic oral prophylaxis and reported no systemic comorbidities.

### 2.1. Clinical and Radiographic Examination

**Figure 1:**
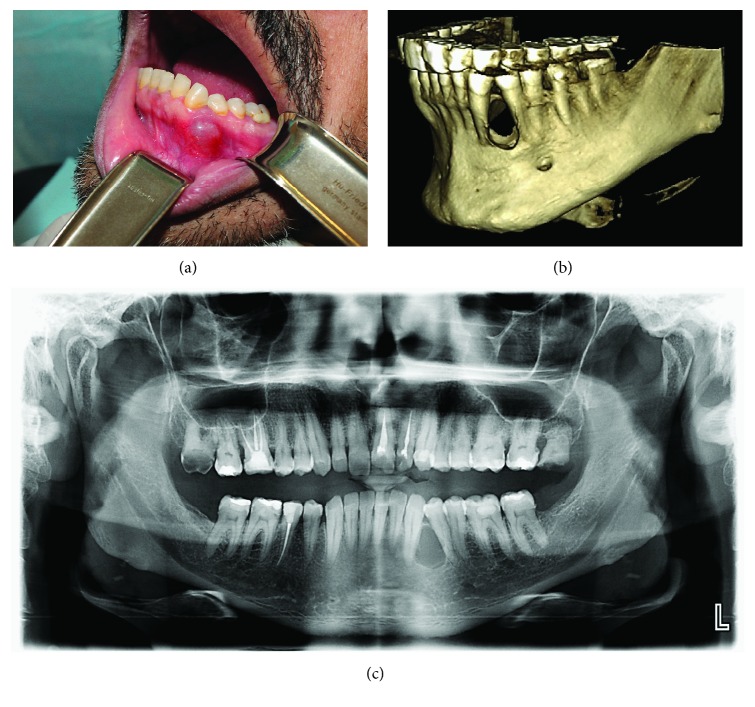
Preoperative clinical photograph and radiographs showing (a) circumscribed swelling in the attached gingiva in between the left mandibular canine (33) and premolar (34); (b) three-dimensional reconstructed cone beam computed tomography image of left mandible showing cystic lesion between roots of teeth 33 and 34, along with loss of buccal and lingual cortices; and (c) orthopantomograph showing well-circumscribed radiolucency with a sclerotic border in between roots of teeth 33 and 34, along with displacement of the roots.

Upon clinical examination, a well-circumscribed and fluctuant swelling, measuring about 9-12 mm in diameter, was observed at the junction of the buccal attached and free gingival margins in between the left mandibular canine (33) and premolar tooth (34) (Figure 1). The swelling was nontender, and there was no dental focus of infection or inflammation in the associated teeth. Similarly, no abnormalities were detected clinically in the lingual aspect of the left mandibular canine and premolar teeth. Both teeth 33 and 34 were vital upon testing with a cold stimulus and upon electric pulp testing. Extra oral examination revealed no clinically discernible asymmetry, swelling, or lymphadenopathy. Aspiration of the swelling with a large bore needle yielded clear fluid indicative of a cystic lesion and ruling out an abscess or vascular lesion.

Radiographic examination (orthopantomogram (OPG)) of the left mandibular body region revealed a well-circumscribed radiolucency, with a sclerotic border in between the roots of teeth 33 and 34. The radiolucency measured approximately 1.5 cm superior-inferiorly and 1.2 cm mesiodistally. Although the roots of the associated teeth (33 and 34) were displaced, there was no discernible loss of the lamina dura in them. Cone beam computed tomography (CBCT) of the region of interest revealed a cystic lesion between the roots of the left mandibular canine and premolar, along with resorption of both the buccal and lingual cortical plates (Figure 1).

### 2.2. Surgical Procedure and Follow-Up

**Figure 2:**
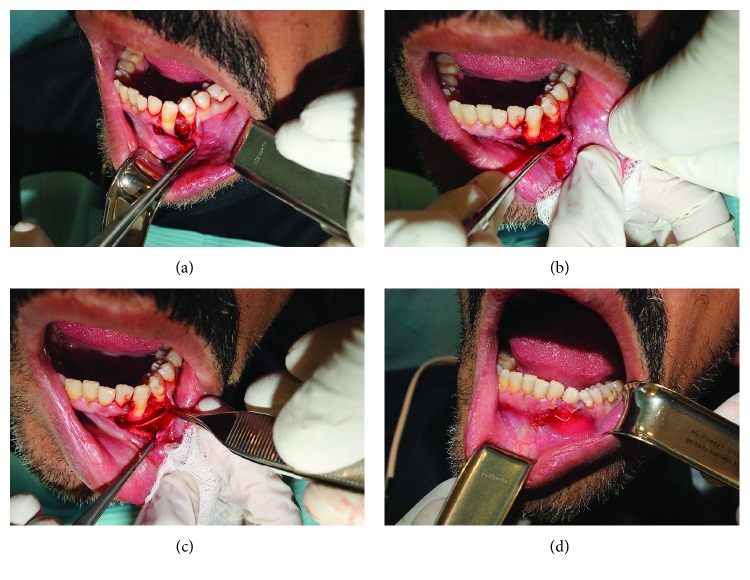
Intraoperative clinical photographs showing (a) mucoperiosteal flap elevation and visualization of the cyst in between roots of teeth 33 and 34; (b) identification and enucleation of the cystic lining; (c) grafting the cyst cavity with xenograft bone and placement of a resorbable collagen membrane; and (d) reapproximation of the mucoperiosteal flap and closure with resorbable sutures.

Based on a clinical and radiographic diagnosis of a lateral periodontal cyst, the patient was consented for enucleation and curettage of the cystic lesion and primary reconstruction of the defect site using guided bone regeneration. Following informed consent, under local anesthesia, the lesion was surgically approached through a gingival crevicular incision extending from tooth 32 to tooth 35. After mucoperiosteal flap elevation, an anterior vertical releasing incision was provided for optimum visualization and sharp dissection using a pair of scissors was done superficial to the cystic wall in order to separate the mucoperiosteal flap without compromising its integrity. While the cystic lesion was identified and enucleated in total, mild adhesions to the lingual mucoperiosteum were released through blunt dissection using the surgical curette (Figure 2).

**Figure 3:**
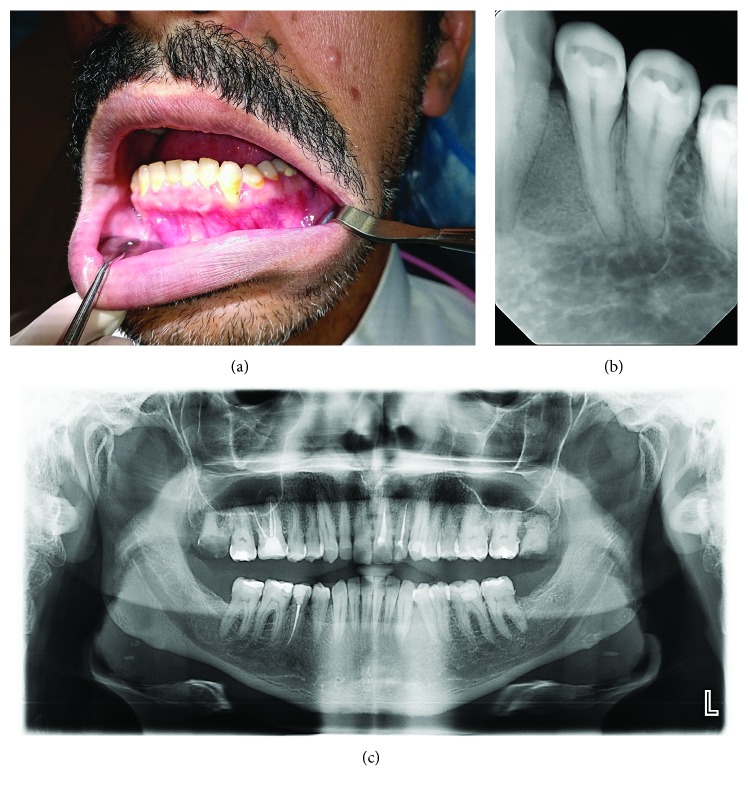
Postoperative clinical photograph and radiographs at one-year follow-up showing (a) healthy gingiva and periodontium in between teeth 33 and 34; (b) periapical radiograph and (c) orthopantomograph showing no evidence of cyst recurrence and complete bone fill between roots of teeth 33 and 34.

Following enucleation and curettage, the cystic cavity was thoroughly irrigated with sterile normal saline. Clinical examination revealed a bony wall with intact mucoperiosteum on the lingual aspect, and there was no resorption of the roots of the tooth mesial and distal to the lesion. The residual defect left behind by the cystic lesion was then filled with a xenograft bone (Bio-Oss, Geistlich Pharma, Princeton, NJ, USA) soaked in sterile normal saline and covered by a resorbable collagen membrane (RCM) (Bio-Gide, Geistlich Pharma, Princeton, NJ, USA). The mucoperiosteal flap was reapproximated and closed with Vicryl 4-0 interrupted sutures (Polyglactin 910, ETHICON, Somerville, NJ, USA) (Figure 2). Postoperative radiographs revealed complete filling of the bone defect with graft material, and the patient was discharged with specific postoperative instructions and antibiotic and analgesic medication. The immediate postoperative period was uneventful, and the surgical wound healed remarkably well. Follow-up examinations at six months and one year after cyst enucleation and guided bone regeneration revealed no clinical or radiographic evidence of recurrence of the lesion. Moreover, teeth 33 and 34 were still vital during follow-up (Figure 3).

### 2.3. Histopathology Examination

**Figure 4:**
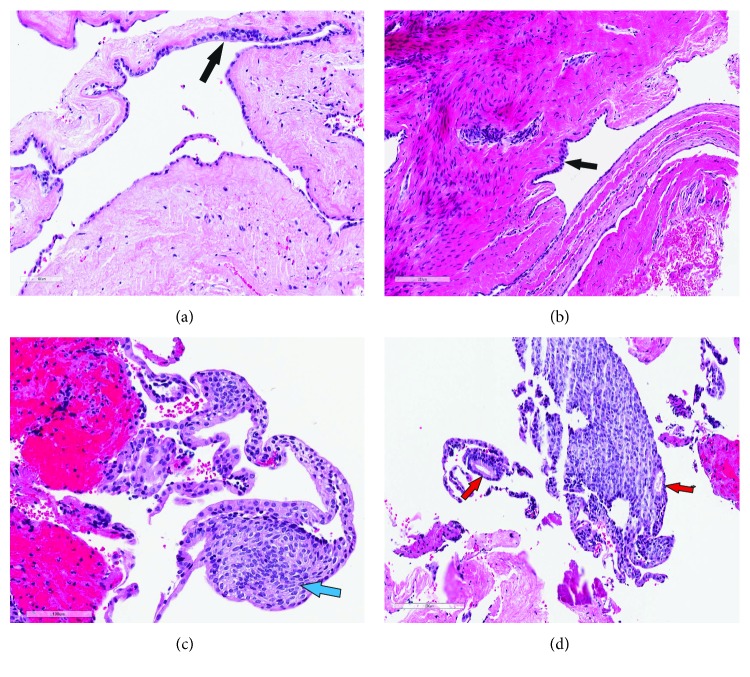
Histopathological examination of (a) the excised cystic lesion showing and (b) cystic lesion composed of reduced enamel epithelium-like lining comprising single or double layer(s) of flattened squamous or cuboidal cells and subtly thickened areas with more closely packed cells (arrow). The cyst wall is uninflamed throughout but demonstrates varying degrees of collagenization and cellularity. Cells with a clear cytoplasm are scattered throughout the lining (a). (c) A fragmented part of the lining showing a thickened cellular plaque with evidence of whorled (swirling) arrangement (blue arrow). Cells with a clear cytoplasm are also seen scattered in this plaque. (d) Another fragmented thickened epithelial plaque with areas showing ductal orientation (red arrow). Scale bars: 100 *μ*m (a, c) and 200 *μ*m (b, d).

The excised soft tissue specimen was immediately fixed in 10% neutral buffered formalin and was sent for histopathology examination (HPE). HPE of the excised specimen revealed a cystic lesion lined in most areas by a thin epithelial lining composed of one or two rows of cuboidal or flattened epithelial cells. Focal areas of epithelial thickenings, with a few duct-like spaces rimmed by cuboidal cells and areas of swirling orientation of the cells, were also noted. Occasional clear cells in the lining along with odontogenic epithelium in the cyst wall were also seen. Based on HPE and clinicoradiological correlation, a diagnosis of lateral periodontal cyst was established. Considering the low risk of recurrence associated with the lesion, it was decided to follow up the patient closely (Figure 4).

## 3. Discussion

In this report, a case of LPC has been presented along with its corresponding clinical, radiographic, and histopathologic findings, in addition to its primary management with GBR. The first documented report of LPC in the literature was as early as 1958 by Standish and Shafer [15], who reported a series of five cases of LPC presenting in the mandibular canine-premolar region. LPC is an uncommon developmental odontogenic cyst which occurs in association with vital teeth and is usually reported as a coincidental finding during routine radiograph [2, 3]. Owing to its noninflammatory nature, it is asymptomatic until and unless the cystic lesion is secondarily infected [3]. In the present case report, the patient did not complain of any symptoms other than a painless swelling in the left mandibular canine-premolar (teeth 33 and 34) region, and the associated teeth were vital. In spite of a clinical swelling, the lesion was coherent with the classical clinical presentation of LPC, namely, asymptomatic swelling associated with vital teeth in the canine-premolar region in a middle-aged patient (Figure 1) [2, 3, 12]. Radiographically, the lesion presented as a well-circumscribed radiolucency surrounded by a sclerotic border and not encroaching upon the periodontal ligament space of the adjacent teeth (Figure 1). The above findings were in line with the radiographic presentation of LPC as reported in the literature [2, 3, 5, 12]. Based on the clinical and radiographic findings, a provisional diagnosis of LPC was considered and treatment was planned accordingly.

In spite of its distinguishing radiographic and clinical presentation, it is considered prudent enough to confirm the diagnosis of LPC through HPE [3]. The characteristic histopathological features of LPC include a nonkeratinized epithelial lining, 1-3 cell layers in thickness, resembling the odontogenic epithelium, along with epithelial plaques and glycogen-rich clear cells [4, 10, 11, 16, 17]. Although the collagen-rich connective tissue wall displays areas of hyalinization, there is no evidence of inflammatory cells [16, 17]. LPC was originally believed to arise from the epithelial rests in the periodontal ligament space, in the absence of any associated inflammatory stimulus [15]. Currently, it is reported to arise from the reduced enamel epithelium, remnants of dental lamina or cell rests of Malassez [3, 5]. It has been hypothesized that the origin of LPC could be correlated to the cell type of origin, based on histopathological findings. While the presence of nonkeratinized epithelium points to an origin from reduced enamel epithelium, evidence of glycogen-rich clear cells indicates the relationship with remnants of dental lamina [3, 18]. On the other hand, the predominantly reported location of LPC close to the root surface implies a possible origin from the cell rests of Malassez [3, 18]. Nevertheless, there is no consensus regarding a particular cell type of origin, as all the abovementioned features have been described by the majority of the cases reported in the literature [3]. In the present case, HPE revealed the presence of a thin layer of nonkeratinized odontogenic epithelium, along with clear cells and epithelial plaques in the cystic lining (Figure 4).

It must however be noted that a HPE finding similar to LPC is also reported in the botryoid odontogenic cyst (BOC) [5, 9]. In contrast to LPC which presents as circumscribed or tear-shaped radiolucency in between roots of teeth, BOC appears as a multilocular radiolucency and presents as a multicystic lesion at both the macroscopic and microscopic levels [3, 4, 9]. It derives its name from the Greek word “botryos,” which means grape-like, and is considered a histopathological variant of LPC with a greater propensity for recurrence following treatment [1, 3, 9, 12]. Although a rare entity, BOC has also been reported to develop from the cystic lining of a preexisting LPC associated with aggressive intrabony expansion [9, 19]. While it is believed that BOC arises as a result of changes in LPC, it has also been reported that BOC has a multicentric origin, wherein several LPCs develop in very close proximity [4, 9, 19]. Nevertheless, there has been a unanimous agreement regarding the common odontogenic origin for both the LPC and BOC [12, 19]. Based on a hypothesis that all LPCs are capable of progressing to multicystic lesions, Altini and Shear [8] proposed a classification of LPC as unicystic, multicystic, or botryoid variants, but all of them with similar histopathological presentation [8]. Interestingly, BOC has also been classified as a “polymorphous odontogenic cyst” along with sialo-odontogenic, glandular, and median-mandibular cysts owing to their predominantly high rates of recurrence [20]. It must therefore be reiterated that HPE to identify any microcysts developing within the LPC lining and wall is required to distinguish the lesion from the aggressive multicystic or botryoid variants [12, 19, 20]. In the present case, there was no histopathological evidence of intraepithelial microcyst formation, thereby confirming the diagnosis of a unicystic LPC variant (Figure 4).

Treatment of unicystic LPC usually involves conservative surgical enucleation of the cyst and curettage of the cystic cavity to eliminate any remnants. In the majority of the cases, the associated teeth remain vital and are neither extracted nor endodontically treated [2, 5, 12]. In the present case too, a similar surgical treatment was carried out. Both the associated teeth (33 and 34) were clinically vital at the beginning of treatment and remained vital and in function after one year of follow-up. However, a primary reconstruction of the cystic cavity was carried out using guided bone regeneration (Figure 2). Similar to our case, in several reported cases of LPC in the literature, enucleation and curettage were done based on a clinical and radiographic diagnosis and confirmation through HPE was done only postoperatively [3, 10, 13, 14, 16–18]. Nevertheless, it is imperative to identify and rule out differential diagnoses of other odontogenic lesions prior to any definitive treatment. Odontogenic cysts including odontogenic keratocyst (OKC) [21], dentigerous cyst [22, 23], and glandular odontogenic cyst (GOC) [24] have mimicked LPC in their presentation. However, the abovementioned odontogenic cysts clinically and radiographically present in association with impacted teeth and are preponderant in the posterior mandibular region [3, 21, 23, 24]. On the contrary, histopathologically confirmed LPC predominantly presented in the periodontal ligament space between the lateral surfaces of teeth, either in the mandibular canine-premolar region or in the maxillary anterior region [2, 5, 12]. Considering the high rates of recurrence associated with lesions such as OKC and GOC, it is justifiable to perform an incisional biopsy of cystic lesions mimicking LPC in the mandibular posterior region.

Even though managed conservatively, enucleation of LPC leaves behind a significant bone cavity and dead space, which in the long term might affect the periodontal health of associated teeth [12]. Several modalities of tissue regenerative techniques have been reported to achieve optimum postoperative periodontal health following surgical treatment of LPC [10, 13]. GBR is routinely used for the treatment of periodontal bone defects and alveolar ridge augmentation prior to implant placement [14], wherein regeneration of an osseous defect is achieved within 6-10 months by placement of an osteoconductive bone substitute material in the defect and covering it with a barrier membrane [14]. While several osteoconductive materials including hydroxyapatite, tri-calcium phosphate, and deproteinized allograft and xenograft are reportedly used in GBR, a resorbable collagen barrier membrane (RCM) is preferred [10, 13, 14]. In addition to preventing fibrous tissue invasion into the osseous defect, RCM enables hemostasis and neovascularization and promotes attachment of osteoblasts [14]. In the present case, a bovine derived deproteinized xenograft bone was used as the osteoconductive scaffold along with RCM and at one-year follow-up, complete bone fill of the defect site was evidenced radiographically (Figure 3). Moreover, healing of periodontal tissues without any complications was also noticed clinically, in between the affected teeth.

## 4. Conclusion

Although LPC is an uncommon developmental odontogenic cyst, it should be considered a differential diagnosis in case of cystic lesions lateral to the surface of a tooth and without any associated inflammatory processes or symptoms. Unicystic LPC clinically and radiographically diagnosed based on the previously mentioned characteristic features can be managed with conservative surgical enucleation and GBR. However, further long-term clinical studies in larger sample sizes should be mandated to ascertain the efficacy of this treatment modality. Nevertheless, the role of HPE after surgical enucleation should not be discounted and histopathological confirmation of diagnosis serves as the benchmark for a recurrence-free follow-up period.

## Figures and Tables

**Table 1:** World Health Organization (WHO) classification of odontogenic tumors and cysts (4th Edition, 2017) [7].

Odontogenic tumors	Malignant tumors	Ameloblastic carcinoma Primary intraosseous carcinoma, NOS Sclerosing odontogenic carcinoma Clear cell odontogenic carcinoma Ghost cell odontogenic carcinoma Odontogenic carcinosarcoma Odontogenic sarcomas
	Benign epithelial origin tumors	Ameloblastoma, conventional (i) Ameloblastoma, unicystic type (ii) Ameloblastoma, extraosseous/peripheral type (iii) Metastasizing (malignant) ameloblastoma Squamous odontogenic tumor Calcifying epithelial odontogenic tumor Adenomatoid odontogenic tumor
	Benign mixed (epithelial-mesenchymal) origin tumors	Ameloblastic fibroma Primordial odontogenic tumor Odontoma (i) Compound type (ii) Complex type Dentinogenic ghost cell tumor
	Benign mesenchymal origin tumors	Odontogenic fibroma Odontogenic myxoma/myxofibroma Cementoblastoma Cemento-ossifying fibroma

Odontogenic cysts	Developmental origin cysts	Dentigerous cyst Odontogenic keratocyst Lateral periodontal and botryoid odontogenic cyst Gingival cyst Glandular odontogenic cyst Calcifying odontogenic cyst Orthokeratinized odontogenic cyst
	Inflammatory origin cysts	Radicular cyst Collateral inflammatory cyst
